# Supplementary material for: Aphelinus nigritus Howard (Hymenoptera: Aphelinidae) Preference for Sorghum Aphid, Melanaphis sorghi (Theobald, 1904) (Hemiptera: Aphididae), Honeydew Is Stronger in Johnson Grass, Sorghum halepense, Than in Grain Sorghum, Sorghum bicolor
Source: Insects. 2022 Dec 22;14(1):10. doi: 10.3390/insects14010010 (PMC9862272; doi:10.3390/insects14010010)
Supplement: Supplementary file 1 [file insects-14-00010-s001.zip › insects-1989970-supplementary.pdf]

Table S1. Proportional Composition of Amino Acids in Grain Sorghum and Johnson Grass Sorghum aphid Honeydew Over Time. Values are mean proportions ± SE of the mean. As per one-way ANOVA, bolded values indicate statistical significance at $p < 0.05$.

	**24 hours**		72 hours		120 hours	
**Amino Acid**	**Grain sorghum**	**Johnson grass**	Grain sorghum	Johnson grass	Grain sorghum	Johnson grass
Alanine	0.09 ± 0.03	0.03 ± 0.03	0.06 ± 0.03	0.1 ± 0.03	0.01 ± 0.04	0.02 ± 0.03
Arginine	0.02 ± 0.02	0.02 ± 0.02	0.07 ± 0.02	0.05 ± 0.02	0.02 ± 0.02	0.01 ± 0.02
Asparagine	0.03 ± 0.01	0.03 ± 0.01	0.03 ± 0.01	0.03 ± 0.01	0.12 ± 0.01	0.11 ± 0.01
Aspartic Acid	0.08 ± 0.02	0.14 ± 0.02	0.01 ± 0.02	0.09 ± 0.02	0.13 ± 0.03	0.11 ± 0.02
Glutamic Acid	0.16 ± 0.03	0.16 ± 0.03	0.11 ± 0.03	0.14 ± 0.03	0.16 ± 0.03	0.15 ± 0.03
Glutamine	0.01 ± 0.01	0.02 ± 0.01	0.02 ± 0.01	0.02 ± 0.01	0.02 ± 0.01	0.17 ± 0.01
Glycine	0.04 ± 0.01	0.03 ± 0.01	0.04 ± 0.01	0.03 ± 0.01	0.01 ± 0.01	0.03 ± 0.01
Histidine	0.01 ± 0.01	0.01 ± 0.01	0.03 ± 0.01	0.01 ± 0.01	0.01 ± 0.01	0.11 ± 0.01
Isoleucine	0.01 ± 0.003	-	0.01 ± 0.003	0.01 ± 0.003	0.01 ± 0.004	0.01 ± 0.003
Leucine	0.01 ± 0.003	0.01 ± 0.002	0.01 ± 0.003	0.01 ± 0.003	0.01 ± 0.004	0.02 ± 0.004
Lysine	-	0.01 ± 0.002	-	-	0.01 ± 0.003	-
Phenylalanine	0.01 ± 0.01	-	0.01 ± 0.01	0.02 ± 0.01	0.01 ± 0.01	0.02 ± 0.01
Proline	0.39 ± 0.07	0.34 ± 0.07	0.31 ± 0.07	0.3 ± 0.07	-	0.02 ± 0.07
Serine	0.07 ± 0.04	0.15 ± 0.04	0.14 ± 0.04	0.12 ± 0.04	0.35 ± 0.04	0.12 ± 0.04
Threonine	0.01 ± 0.001	0.02 ± 0.006	0.02 ± 0.006	0.04 ± 0.006	0.03 ± 0.007	0.01 ± 0.006
Tryptophan	0.01 ± 0.003	-	0.01 ± 0.003	-	0.02 ± 0.003	**0.09 ± 0.003**
Tyrosine	**0.02 ±0.003**	0.003 ± 0.003	0.02 ± 0.004	0.01 ± 0.004	0.04 ± 0.004	0.03 ± 0.004
Valine	0.02 ± 0.01	0.02 ± 0.01	0.01 ± 0.01	0.02 ± 0.01	0.01 ± 0.01	0.01 ± 0.01
